# Real-Time Selective Markerless Tracking of Forepaws of Head Fixed Mice Using Deep Neural Networks

**DOI:** 10.1523/ENEURO.0096-20.2020

**Published:** 2020-06-05

**Authors:** Brandon J. Forys, Dongsheng Xiao, Pankaj Gupta, Timothy H. Murphy

**Affiliations:** 1 Department of Psychiatry, Kinsmen Laboratory of Neurological Research, University of British Columbia, Vancouver, British Columbia V6T 1Z3, Canada; 2 Department of Psychology, Djavad Mowafaghian Centre for Brain Health, University of British Columbia, Vancouver, British Columbia V6T 1Z4, Canada

**Keywords:** closed loop, movement tracking, real-time tracking, DeepLabCut

## Abstract

Here, we describe a system capable of tracking specific mouse paw movements at high frame rates (70.17 Hz) with a high level of accuracy (mean* *=* *0.95, SD* *<* *0.01). Short-latency markerless tracking of specific body parts opens up the possibility of manipulating motor feedback. We present a software and hardware scheme built on DeepLabCut—a robust movement-tracking deep neural network framework—which enables real-time estimation of paw and digit movements of mice. Using this approach, we demonstrate movement-generated feedback by triggering a USB-GPIO (general-purpose input/output)-controlled LED when the movement of one paw, but not the other, selectively exceeds a preset threshold. The mean time delay between paw movement initiation and LED flash was 44.41 ms (SD* *=* *36.39 ms), a latency sufficient for applying behaviorally triggered feedback. We adapt DeepLabCut for real-time tracking as an open-source package we term DeepCut2RealTime. The ability of the package to rapidly assess animal behavior was demonstrated by reinforcing specific movements within water-restricted, head-fixed mice. This system could inform future work on a behaviorally triggered “closed loop” brain–machine interface that could reinforce behaviors or deliver feedback to brain regions based on prespecified body movements.

## Visual Abstract

**Figure:**
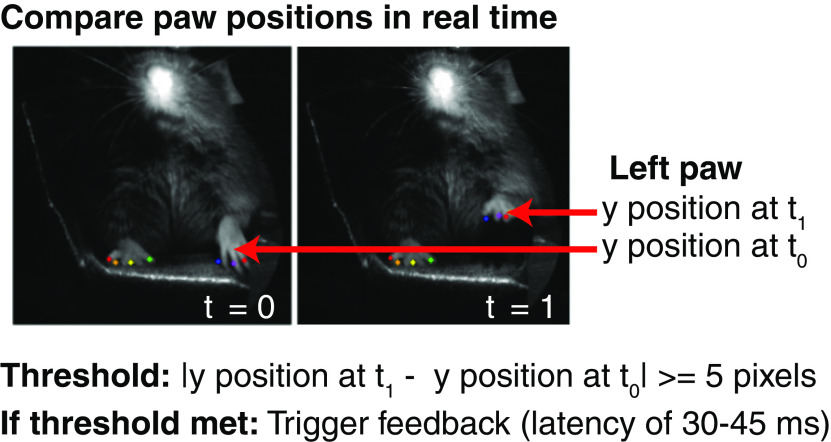

## Significance Statement

We present a software and hardware scheme modified from DeepLabCut—a robust movement-tracking deep neural network framework—which enables real-time estimation of paw and digit movements of mice. Coupled to the body part tracking is the ability to rapidly trigger external events such as rewards on the detection of specific behaviors. This system lays the groundwork for a behaviorally triggered “closed loop” brain–machine interface that could reinforce behaviors and deliver feedback to brain regions based on prespecified body movements.

## Introduction

The accurate quantification and manipulation of behavioral dynamics of animals is important for understanding the neural basis of motor function (Jin and Duan, 2019; Moreira et al., 2019). Real-time movement tracking is a challenging computer vision problem that is crucial for constructing precise movement-triggered feedback systems and brain–machine interfaces needed for mechanistic studies of animal behavior. Furthermore, real-time movement tracking and feedback would enable rapid reinforcement of user-defined behaviors of interest. Significant progress in the development of movement-tracking technology enables accurate pose estimation in humans (Insafutdinov et al., 2016) and animals (Mathis et al., 2018) without the need to manually label large datasets as inputs for training. In particular, with regard to tracking animal movement, the approach presented by the DeepLabCut toolbox of Mathis et al. (2018) generalizes well across morphologically diverse animals. Traditional methods of tracking movement are often based on large databases of stereotyped movement data, such as those used by Insafutdinov et al. (2016), for pose estimation. In contrast, the DeepLabCut approach (Mathis et al., 2018) enables users to generate models that can be more sensitive to movements of a variety of animals using a smaller dataset. While DeepLabCut is a robust tool for pose estimation (Mathis et al., 2018), it has primarily been used for *post hoc* analysis of behavior and not in real time. A customizable framework for real-time tracking of specific body parts in target subjects would have many applications in psychiatry, rehabilitation engineering, and other fields where closed-loop feedback is used. An interesting application and future work direction would be to investigate cortical regions involved in the coordination and planning of movements when used in combination with optogenetics (Ayling et al., 2009; Guo et al., 2015; Mathis et al., 2017). In addition to rapid feedback, real-time analysis would permit behavioral reinforcement by coupling specific movements to reward.

To use high-resolution video and markerless pose estimation as an input for real-time feedback, we require a robust system that can process and track individual body parts with low latency. However, the majority of low-latency real-time tracking systems are based on blob detection algorithms (de Chaumont et al., 2019; Kim et al., 2018), or rely on *post hoc* classification of movement (Gabriel et al., 2019) or highly specialized behavioral arenas that collect data through multiple modalities (Moreira et al., 2019). Such methods, while often less computationally intensive than pose estimation algorithms, are better suited to whole-body tracking than body part tracking. The few markerless tracking systems that do exist typically require extensive, specialized body part detection logic (Sehara et al., 2019; Mathis and Mathis, 2020), rendering them relatively inflexible across different animals. As such, while previous approaches to real-time tracking are effective for examining social interactions or holistic body movements, they are typically unable to selectively discern small-scale movements, such as whisker, digit, or nose movements. We present adaptations to DeepLabCut (Mathis et al., 2018) to leverage it for real-time movement tracking and analysis based on the conditional movement of individual body parts in head-fixed mice with latencies that averaged 44 ± 36 ms (study 1). Additionally, we validate our real-time feedback system by using it to train a group of water-restricted, head-fixed mice to make user-defined forepaw movements (study 2).

## Materials and Methods

Animal protocols (A13-0336 and A14-0266) were approved by national use guidelines. Animals were housed in a vivarium on a 12 h day/light cycle (7:00 A.M. lights on). For head fixation hardware surgery (Silasi et al., 2016), animals were anesthetized with isoflurane (2% in pure O_2_) and body temperature was maintained at 37°C using a feedback-regulated heating pad monitored by a rectal thermometer while they received a cranial window. Mice received an intramuscular injection of 40 μl of dexamethasone (2 mg/ml) and a 0.5 ml subcutaneous injection of a saline solution containing buprenorphine (2 μg/ml), atropine (3 μg/ml), and glucose (20 mm), and were placed in a stereotaxic frame. After locally anesthetizing the scalp with lidocaine (0.1 ml, 0.2%), the skin covering the skull was removed and replaced by dental cement (Ayling et al., 2009; Hira et al., 2009; Silasi et al., 2016). A metal screw was attached to the chamber for future head fixation during experiments. At the end of the procedure, the animal received a second subcutaneous injection of saline (0.5 ml) with 20 mm glucose and recovered in a warmed cage for 30 min.

For the first study, head-fixed mice (male) were stabilized by attaching a skull-mounted screw to a pole mounted on a baseplate while the body was resting in a tube (Silasi et al., 2016). We attach an LED to this pole so that it is visible in the video recording of the mouse, enabling ground-truth validation of the behavioral feedback paradigm by quantifying the resulting light flash.

For a second study investigating our system’s ability to automatically reinforce mice for making specific movements, mice (male, *n* = 8) underwent head-bar surgery and were trained to learn a movement-related task under a head-fixed condition. Before starting the behavioral training, *ad libitum* access to water was stopped. Mice were handled daily and received 1 ml of water per day until they reached ∼85% of initial weight (typically 5–7 d after the start of water restriction). During the handling period, mice were habituated to the experimental setup. The duration of head fixation was progressively increased at a rate of 5 min/d. Handling and head restraining were performed with care taken to minimize the discomfort of the animals. For the task, these mice were head fixed and positioned as in the first study. In addition to the setup described in the first study, a waterspout was placed **∼**3 mm in front of the mouse’s mouth, such that the mouse could only acquire water after making a movement with its paw that was quantified by real-time tracking.

**Figure 1.**
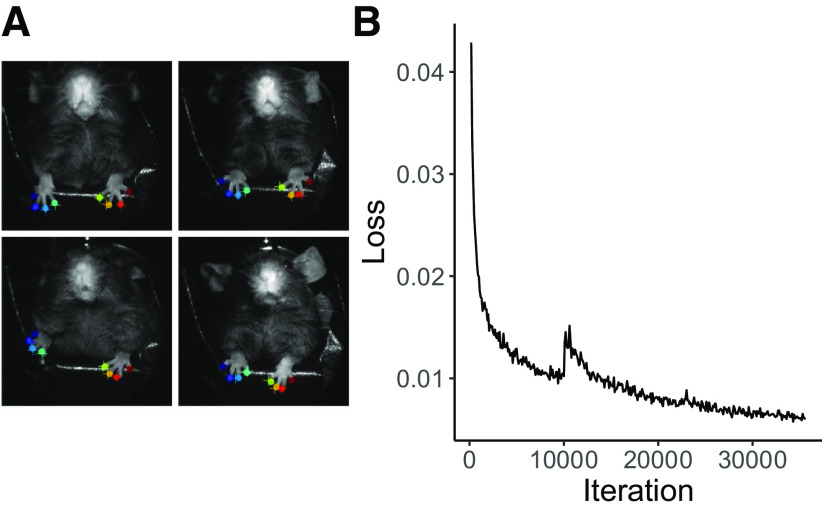
Automated labeling of paws using DeepLabCut for near real-time tracking. ***A***) A comparison of human labels (circles) and DeepLabCut’s automated labels (crosses) for both paws across four different mice in the testing set of the model. ***B***) The loss of our model converged near zero after approximately 30,000 iterations.

*Training movement-tracking models.* We use DeepLabCut 2.0.6 (Mathis et al., 2018) as the basis for our movement-tracking framework. A general model of mouse paw movement was trained based on ResNet-50 by labeling 200 frames selected using k-means clustering (Nath et al., 2019) to sample a variety of movement dynamics from one video of each of the 10 mice recorded (*N *=* *10 videos). These videos were recorded using an Omron Sentech STC-MCCM401U3V USB3 Vision camera through the StCamSWare software (Omron Sentech) at a resolution of 256 × 256 pixels. To generate this model, we labeled the tips of all eight toes on both forepaws in videos of mice recorded in a similar environment and lighting conditions as the environment for the final behavioral trials for each mouse. We trained our model for 35,000 iterations, with a 95% train–test split (Fig. 1). All image processing, tracking, and LED output were conducted on an ASUS All Series computer running Windows 8.1 with 64 GB of RAM, 3.4 GHz, and an Nvidia Titan Xp GPU.

**Figure 2.**
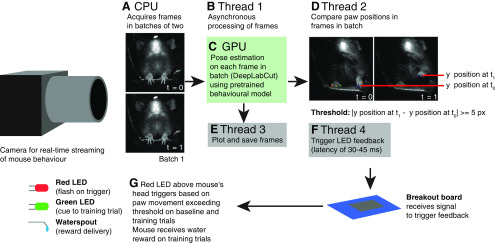
Outline of the acquisition and processing pipeline for selective paw tracking and behavioral triggering. ***A***) Frames are acquired from the camera in batches of two at a time, to enable the direct comparison of movement from one frame to the next. The two frames are passed to a processing thread ***B)*** using Python’s _thread library to allow this batch of two frames to be processed simultaneously with the other frames. ***C***) The frames are analyzed for locations of the body parts identified in the user-trained DeepLabCut machine learning model using GPU acceleration, outputting the coordinates of the paw positions. ***D***) The positions of each of the four paws on the mouse’s toes are averaged across each paw; this averaged paw position is used to compare the paw position between the first and second frames of the two-frame batch. If the absolute vertical movement of the left paw equals or exceeds 5 pixels while the vertical movement of the right paw does not exceed 10 pixels, a signal is sent to the GPIO breakout board to trigger the LED. While each frame – labelled with the predicted body part locations – is saved in a separate thread ***E***) to limit computational load on the main thread, the LED feedback itself is sent in another thread ***F***) to ensure that the feedback can be delivered asynchronously relative to the rest of the code. ***G***) The LED, which is attached to the head-fixing pole above the mouse’s head, is illuminated, demonstrating the procedure of feedback to the animal. On training trials, a water reward is also delivered to the animal at this time.

*Real-time tracking.* We stream a video of each mouse (*N *=* *10) to the desktop computer using an Omron Sentech STC-MCCM401U3V USB3 Vision Camera (Omron Sentech) at a resolution of 256 × 256 pixels for 130 s (Fig. 2*A*).

To allow the frame rate and computations of DeepLabCut to stabilize, we run the movement analysis, but do not save the analysis or provide feedback, for the first 10 s of each trial; as such, we record 120 s of movement and feedback data for each trial. This 10 s buffer period allows a large number of threads that are necessary for rapid computations to start. The base version of DeepLabCut is optimized to process existing video frames within batches at a high speed. When tracking and reinforcing animal behavior in near real time, we do not have the luxury of letting several frames pool into a batch before conducting simultaneous pose estimation and behavioral feedback on all frames in the batch, as this would delay feedback to the animal that is dependent on the pose estimation. As such, we prioritize faster processing of smaller batches to rapidly deliver feedback to the animal based on a guaranteed comparison between consecutive frames. The best strategy we were able to find to accomplish this rapid processing is the simultaneous creation of numerous parallel threads that take in video input, estimate the pose of the animal, and output the feedback signal. For every batch of two frames from the camera, our system creates four threads (Fig. 2). These threads are shut down once they are finished; however, for the first 10 s, the number of threads created exceeds the number of threads being destroyed. After the first 10 s, threads are created and destroyed at a relatively stable rate, easing computational load and improving the stability of the frame rate. We found that 10 s was the minimum buffering time required to achieve a stable frame rate for the rest of the task.

To test the performance of the tracking and feedback at various frame rates, we set the input frame rate of the camera to 90 Hz for 13 trials, 180 Hz for 13 trials, 200 Hz for 13 trials, 220 Hz for 7 trials, 270 Hz for 14 trials, 300 Hz for 11 trials, 320 Hz for 9 trials, and 360 Hz for 1 trial (Table 1). We varied the frame rate of the camera from 90 to 360 Hz and assessed the specificity of tracking. A range of frame rates was tried with some higher frame rates showing errors, and 200 Hz offered an optimal compromise between tracking efficiency and trigger latency. Two trials—one recorded at 220 Hz and one recorded at 360 Hz—were excluded because of program runtime issues leading to mean delays of >500 ms. One trial recorded at 270 Hz was excluded because the mouse did not move enough to exceed the minimum movement threshold, resulting in no triggers above criterion for this trial. There were 27 trials in which the mouse briefly engaged in grooming behavior; while this resulted in behavioral triggers being sent, it did not affect the tracking accuracy or delay of feedback. The shutter speed of the camera was set to one-five hundredth of a second for all trials. We use the pysentech library (https://github.com/derricw/pysentech) to allow Python to interface with the camera. As each video frame arrives on the computer, we convert it to an 8 bit unsigned byte format and pass it to the pose analysis function in DeepLabCut in a batch of two consecutive frames, in a separate thread for each batch (Fig. 2*A*). This function returns the predicted positions of each toe on the left paw of the mouse. In an additional thread (Fig. 2*E*), we render these coordinates onto the newly analyzed frame using opencv2 (https://opencv.org/) to enable visual inspection of tracking quality.

**Table 1:** A data table for all trials run in the first study

Input frame rate (Hz)	Mean output frame rate (Hz)	SD output frame rate (Hz)	Mean delay (ms)	SD delay (ms)	Mean accuracy	SD accuracy	*n*
90	46.95	1.53	34.34	5.94	0.97	0.01	13
180	68.13	5.10	39.85	42.97	0.94	0.02	13
200	70.17	7.07	32.56	10.70	0.95	0.00	13
220	69.31	0.33	33.82	15.10	0.95	0.03	6
270	80.83	18.27	55.93	55.69	0.94	0.01	13
300	91.45	5.27	60.62	77.71	0.94	0.01	11
320	109.76	49.24	53.28	43.80	0.93	0.04	9

Input frame rate, the streaming frame rate set in the software for our camera, representing the frame rate of the camera without any additional analyses; mean output frame rate, the mean number of frames per second processed by our system across all recordings at each input frame rate; SD output frame rate, the SD in the number of frames per second processed by our system across all recordings at each input frame rate; mean delay, the mean amount of time, in milliseconds, between the system receiving a frame that contains a left paw movement exceeding 5 pixels of vertical movement and the system sending a signal to the breakout board to trigger the LED to provide feedback for that movement; SD delay, the SD, in milliseconds, of the delay discussed above; mean accuracy, the mean output of the sigmoid function by TensorFlow for all body parts for all trials at each frame rate; SD accuracy, the SD of the output of the aforementioned sigmoid function; *n*, the number of trials recorded at each frame rate.

*Real-time feedback.* In order to deliver feedback based on specific paw movements, we define a target paw movement to reinforce. We operationalize this paw movement as a difference in the average vertical position of the four estimated toe positions on the left paw of the mouse from one frame to the next that is greater than a minimum threshold but smaller than a maximum threshold that we define. These thresholds ensure that small shifts in the mouse paw, or large errors in tracking, do not result in erroneous feedback to the animal. We chose to track the vertical movement of the left paw of the mouse because this movement approximates a reaching activity, which is an example of a movement that could be conditioned using our feedback paradigm. Based on the frame resolution and the paw movements of our mouse, we set the minimum threshold as 5 pixels of vertical movement of the left paw of the mouse, and the maximum threshold as 100 pixels of vertical movement of this paw (to prevent feedback being delivered if the tracking erroneously jumps across the screen). To help ensure that the feedback was selective for left paw movement, no feedback was delivered if the right paw of the mouse exceeded 10 pixels of vertical movement, regardless of left paw movement (Fig. 2*D*). We set the right paw movement limit threshold as twice as large as the threshold for the left paw because we found that the initial group of mice that we assessed was prone to making more spontaneous right paw movements than left paw movements. A strict restriction of 5 pixels on both paws (right paw no more than 5 pixels and left paw at least 5 pixels) was associated with an overall decreased number of triggers as even small, unreinforced right paw movements often occurred at the same time as left paw movements. Importantly, the software we provide is flexible, and the criterion can be set to reflect the movement of any set of tracked points. If the vertical movement of the left paw from one frame to the next is between 5 and 100 pixels inclusive, we give visual feedback using a trigger set by the pyftdi library (https://github.com/eblot/pyftdi) to turn on a red LED on the head-fixing pole for 200 ms over USB via an Adafruit FT232h Breakout Board (Adafruit Industries; Fig. 2*G*). As a further safeguard against erroneous triggering of feedback, this feedback is triggered only if DeepLabCut determines that the body part prediction accuracy for that frame (quantified as the output of the TensorFlow sigmoid function) is >0.20. Last, to ensure that feedback is not continuously delivered to the mouse in the event of repeated paw movement above threshold, we set a refractory period of 300 ms after each trigger during which no trigger is delivered regardless of movement dynamics (for three mice across 21 trials).

In a second study, we validated the ability of our method to assess and reinforce a user-defined behavior in real time. Mice were presented with alternating baseline and training trials (*n *=* *115 baseline; *n* = 115 training) across 5 d. Each of these trials had the same length, structure, and computational analyses of behavior used in the first study. Mice were cued to training trials by the illumination of a green LED located **∼**20 mm to the left and above the head of the mouse, for the duration of the trial. On training trials, the mouse received water for 150 ms from the waterspout in front of its mouth if it made a criterion forelimb movement (≥5 pixels of vertical movement of the left paw while simultaneous movement on the right paw was <10 pixels). In this study, the mouse was not rewarded for making right paw movements exclusively, and making a right paw movement following a left paw movement increased the probability of not receiving a reward. The use of the criterion of the 10 pixel right paw movement limit increased the ability of our system to reward selective left paw movements. This water reward was accompanied by the illumination of a red LED located **∼**20 mm to the left and above the head of the mouse for 200 ms. In comparison, on baseline trials, the mouse did not receive feedback for making a criterion forelimb movement, and the green LED did not illuminate for the length of the trial. However, the red LED continued to flash when the mouse made the criterion movement. In order to have a consistent ground-truth measure of the mouse making the reinforced movement, trials were recorded using the same methodology as outlined in the first study. All trials in the second study were recorded at 200 Hz, as this was the frame rate that offered the best combination of a high frame rate and low feedback latency in the first study (Table 1).

*Data availability.* We provide a Python script to enable real-time tracking within the existing DeepLabCut 2 workflow, and a modified set of pyftdi classes to allow interfacing between Python and the general-purpose input/output (GPIO) board. This code is freely available online at https://github.com/bf777/DeepCut2RealTime. We also provide a MATLAB script to enable automatic analysis of behavior videos collected in real time. This code is freely available online at https://github.com/DongshengXiao/RealTimeTracking. We provide copies of this acquisition and analysis software online as Extended Data 1 and 2 files.

10.1523/ENEURO.0096-20.2020.ed1 Extended Data 1 Supplementary LED analysis code. Download LED analysis code, ZIP file

10.1523/ENEURO.0096-20.2020.ed2 Extended Data 2 Supplementary DeepCut2RealTime code. Download DeepCut2RealTime code, ZIP file

## Results

### Behavioral model

The behavioral model that we trained to predict the movements of the mouse in DeepLabCut had a root mean squared error of prediction of 2.17 pixels for the training data and 2.37 pixels for the test data when using a standard scene that averaged 44 × 44 mm (visualized in Fig. 1*A*).

### Real-time tracking

We recorded a total of 81 trials across nine mice, with each trial being 130 s in length. A total of 78 trials across these nine mice were analyzed (for exclusion explanation, see Materials and Methods). We quantified the behavioral tracking quality of each trial by calculating the average frame rate over 120 s (after the conclusion of the 10 s buffer period), and the average accuracy of each prediction in each frame being correct as measured by the TensorFlow sigmoid function as implemented in DeepLabCut. The mean tracking accuracy across all trials was 0.95 (SD* *=* *0.01). Input frame rates were much higher than the output frame rates, suggesting that some frames were dropped. For example, an input frame rate of 200 Hz would yield an output rate of the frame rate of 70 Hz. While the dropping of frames is concerning, we do record the frame times for the reconstruction of time courses. Lower frame rates of 90 Hz dropped fewer frames, leading to an output frame rate of ∼45 Hz (Table 1).

### Real-time feedback

**Figure 3.**
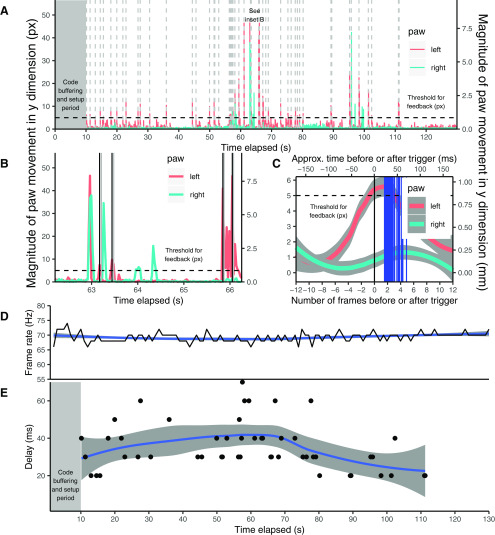
Selective paw movements tracked with short latency and coincident with behavioral feedback triggering. ***A***) Magnitude of movement in the vertical direction for the mouse’s left and right paws across one trial, recorded with an input frame rate of 220 Hz and having an output frame rate of 70 Hz. The dotted vertical lines represent times in the trial when the mouse’s left paw movements exceeded the threshold, triggering feedback via the LED. ***B***) Detail of the mouse’s vertical paw movements over a period of 280 frames. The solid black lines indicate the time at which the frame during which the mouse’s movements crossed the threshold was sent from the camera; the red region and lighter grey line represent the time between frame acquisition and the LED being triggered as feedback. ***C***) Magnitude of movement in the vertical direction for the mouse’s left and right paw for each trigger in the trial depicted in A), averaged for each frame before and after the trigger. Each solid blue line represents a single trigger at the actual time of the trigger, depicting the delay between the time at which the frame containing the above-threshold movement was received and the time at which the command was sent to activate the LED trigger. ***D***) Dynamics of output frame rate for each second during the trial; the background curve is a Loess curve showing the smoothed average frame rate at that moment. ***E***) Time of each behavioral trigger during the trial, plotted against the delay between the time at which the frame containing the above-threshold movement was received and the time at which the command was sent to activate the LED trigger. Note: For all plots with movement distance in mm, this distance in mm was calculated by measuring the width of the floor of the mouse’s tube and cross-referencing this distance with the width of the floor of the mouse’s tube in a frame recorded from this trial, in pixels. For plot ***C***) the time elapsed in milliseconds is an approximate measure because of slight variations in the number of frames processed per second across the trial (as highlighted in ***D***).

To evaluate the delay between frame acquisition and the LED flash that signaled a movement that reached criterion, we record the time at which the frame was sent from the camera, the time at which the feedback criterion was met, and the time at which the LED turned on based on the collected video that contains the behavioral video of the mouse. Plotting the movement of the left and right paws for trials where trigger conditions were met indicated—as expected—a preference for left paw over right paw movement (Fig. 3). Importantly, the LED trigger was delivered within ∼40 ms of the left paw crossing the threshold, which was estimated to be a 0.85 mm movement. We quantified the feedback latency as the average delay between frame acquisition and feedback delivery on trials. This delay was measured by time stamps from Python where movement of the left paw exceeded the minimum threshold and was below the maximum threshold while—simultaneously—movement in the right paw was below the maximum threshold for that paw. Across all trials, the mean delay between movement initiation and LED illumination across trials (*N = *9 mice, 78 trials) was 44.41 ms (SD* *=* *36.39 ms[Fig F4]). Summary data broken down by input frame rate are outlined in Table 1. We depict typical behavioral reinforcement of the mouse in Movie 1.

**Figure 4. F4:**
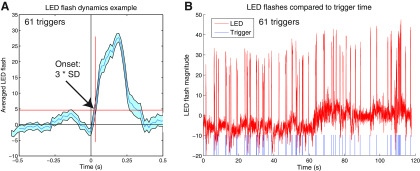
Example of contrast changes from LED flash during one trial. ***A***) Example of contrast changes from LED flash during one trial. Y axis represents magnitude of contrast level in a user-defined region of interest surrounding the LED relative to baseline, averaged across all trials in which contrast increased by at least three standard deviations above baseline. ***B***) Example of all contrast changes from LED flash during one trial. Y axis represents magnitude of contrast level in a user-defined region of interest surrounding the LED relative to baseline, averaged across all trials in which contrast increased by at least three standard deviations above baseline. Each blue tick (“trigger”) represents a time at which the mouse made a vertical left-paw movement greater than or equal to 5 px while the right paw did not move more than 10 px vertically.

Movie 1. This video presents a recording of a mouse's behavior during a training session on the fifth and final day of training. It was created from frames automatically labelled with the position of each digit on each forepaw that were saved during the session, displayed at a frame rate of 30 Hz for clarity. The frames represent 2.90 s of real time recording (at an average frame rate of 65.76 Hz). The lower of the two LEDs in the top right of the image illuminates at the same time as the delivery of a water reward through the spout in front of the mouse's mouth. When there is a set of movements in rapid succession, the LED does not flash for every single movement because it operates with a 300 ms refractory period. This period ensures that the mouse is not reinforced for making a very large quantity of small movements as opposed to discrete, larger movements. 10.1523/ENEURO.0096-20.2020.video.1

In order to provide a second ground-truth measure of feedback latency and verify these delay values, we recorded a second set of trials (*N *=* *5) with a 200 Hz frame rate in which the LED illumination was readily visible within the video frame, offering a visual indicator of the feedback onset. We used MATLAB (version 2014b; MathWorks) to define a region of interest surrounding the LED in each recording and quantified each LED illumination as a change in pixel value >3 SDs from the mean background. As an increased number of frames were dropped within the first 800 frames of each trial (∼4 s; Fig. 3) we quantified the LED illumination time after the 800th frame. We then quantify ground-truth latency as the time between the start of the frame on which a significant movement was detected and the point at which the LED illumination crosses the criterion value discussed above. The mean ground-truth latency calculated through this method was 35.90 ms; the mean latency calculated through the time stamps in the code was 32.40 ms. We found no significant difference between the ground-truth latency and the time-stamp latency (*t*_(4)_ = 0.76, *p *=* *0.54). The average waveform of the LED flash for an example trial is given in Figure 4*A*; the overall waveform for the LED flash is plotted alongside behavioral triggers in Figure 4*B*.

**Figure 5.**
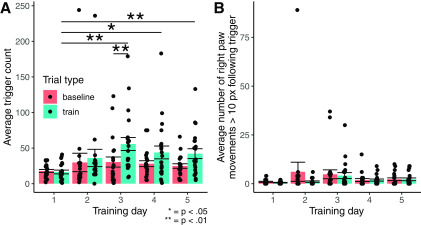
Average number of real-time behavioral triggers on baseline and training trials. Error bars represent standard errors of the mean. ***A***) Average number of triggers represents the average number of times, per trial, that animals made a left paw movement greater than or equal to 5 pixels while not simultaneously making a right paw movement greater than 10 pixels. ***B***) Average number of triggers represents the average number of times, per trial, that animals made a right paw movement greater than 10 pixels on the frame immediately after the animal made a left paw movement greater or equal to 5 pixels (while not simultaneously making a right paw movement greater than 10 pixels). On training trials, each trigger was associated with a water reward and flash of the red feedback LED. On baseline trials, each trigger was only associated with a flash of the red feedback LED. We used paired-samples t-tests that were Bonferroni corrected for multiple comparisons to evaluate between-day differences in average trigger count during training on the first day to average trigger counts across all training sessions on all other days. We also used these tests to evaluate within-day differences in average trigger count between training and baseline trials on each day.

For our second study, in which we reinforced mice for making movements that exceeded the criterion discussed for the first study, we analyzed 5 d of training from seven mice. Although we had eight mice total, we excluded one mouse from our analyses as that mouse did not run on all 5 d. We analyzed a total of 102 baseline and 102 training trials (21 trials of each type per day except on the second day of training, where there were 18 trials of each type). For the summary data for the second study (Table 2), we only excluded one training trial of one mouse that did not make any movements above criterion. Comparing the average number of movement triggers made for baseline and training trials allowed us to quantify the overall success of our automated reinforcement paradigm at motivating the forelimb movement behavior of the mouse (Fig. 5*A*). Based on paired-sample *t* tests with Bonferroni correction for multiple comparisons between training trials for each day and training trials on the first day, the average number of above-threshold movements was significantly greater in training trials on the third, fourth, and last days of training compared with training trials on the first day of training (Fig. 5*A*). There were no significant differences in above-threshold movements between baseline trials for each day and baseline trials on the first day. Based on paired-sample *t* tests with Bonferroni correction for multiple comparisons between training trials for each day and baseline trials for each day, there was a significant difference in above-threshold movements between training and baseline trials on the third day. In order to further validate the selectivity of our system for left paw movement, we evaluated the number of right paw movements >10 pixels (the maximum criterion for right paw movement to receive a reward) for each frame immediately following a successful left paw trigger (Fig. 5*B*). Based on paired-sample *t* tests Bonferroni correction for multiple comparisons, we found no significant differences between days or between baseline or training trials in the number of right paw movements >10 pixels immediately following above-threshold left paw movements. Across all trials, the average number of these right paw movements >10 pixels was lower than the average number of immediately preceding left paw movements ≥5 pixels.

**Table 2:** A data table for all trials run in the second study

Day	Input framerate (Hz)	Mean outputframe rate (Hz)	SD outputframe rate (Hz)	Mean delay(ms)	SD delay(ms)	Meanaccuracy	SDaccuracy	*n*
1	200	65.00	0.53	37.00	10.30	0.99	0.00	21
2	200	66.70	18.26	39.50	10.81	0.94	0.01	20
3	200	66.05	0.65	29.69	7.01	0.90	0.04	24
4	200	65.89	0.57	31.03	7.50	0.95	0.02	24
5	200	65.76	0.54	30.96	8.34	0.95	0.03	21

Input frame rate, the streaming frame rate set in the software for our camera, representing the frame rate of the camera without any additional analyses; mean output frame rate, the mean number of frames per second processed by our system across all recordings at each input frame rate; SD output frame rate, the SD in the number of frames per second processed by our system across all recordings at each input frame rate; mean delay, the mean amount of time, in milliseconds, between the system receiving a frame that contains a left paw movement exceeding 5 pixels of vertical movement and the system sending a signal to the breakout board to trigger the LED to provide feedback for that movement; SD delay, the SD, in milliseconds, of the delay discussed above; mean accuracy, the mean output of the sigmoid function by TensorFlow for all body parts for all trials at each frame rate; SD accuracy, the SD of the output of the aforementioned sigmoid function; n, the number of trials recorded at each frame rate.

**Figure 6.**
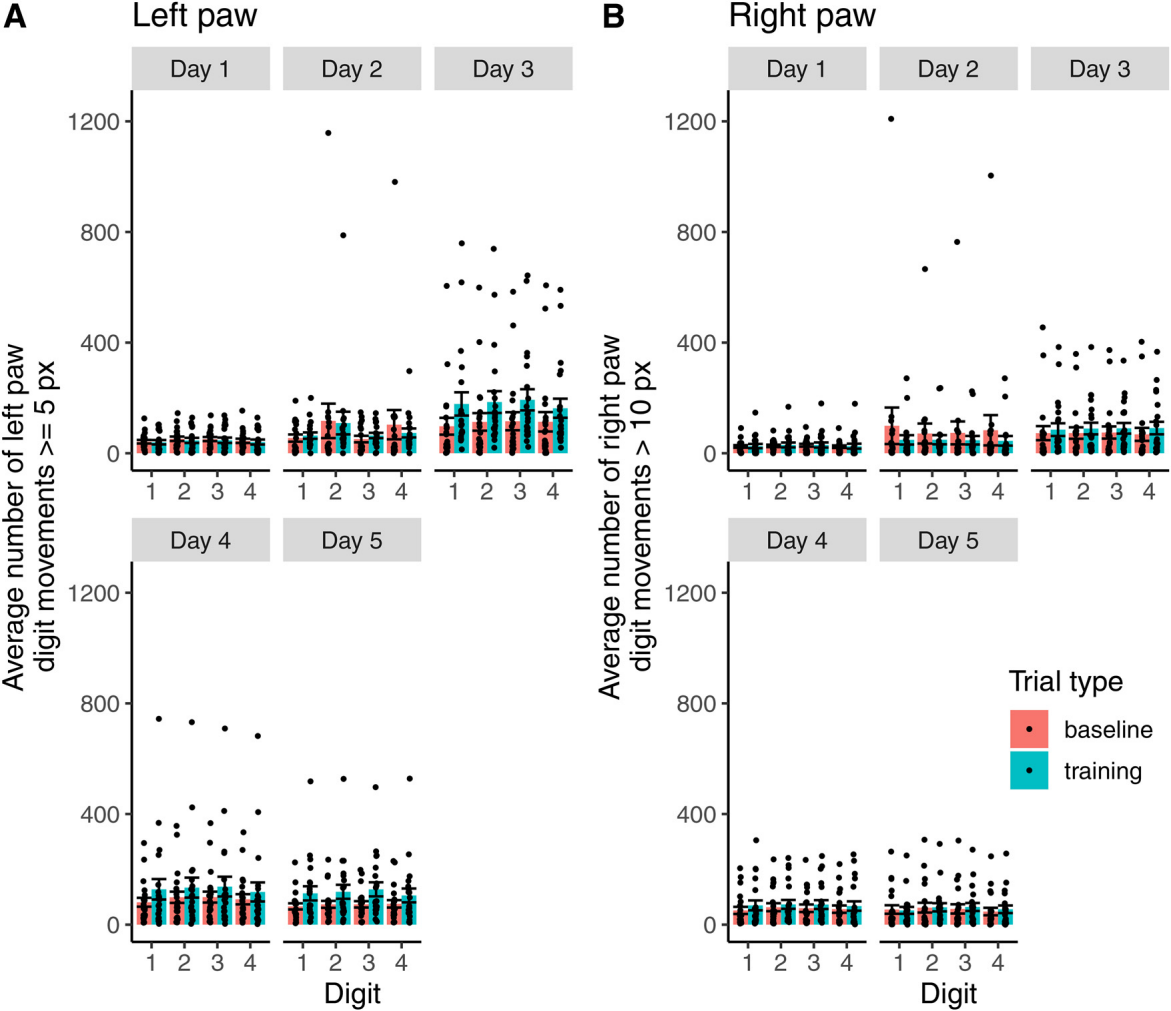
Average number of reaches above threshold on baseline and training trials by digit. ***A***) The average number of vertical left-paw digit movements ≥5 px by digit on training and baseline trials. ***B***) The average number of vertical right-paw digit movements > 10 px by digit on training and baseline trials. Digits are numbered from the rightmost to leftmost digit (from the mouse’s perspective) on each forepaw. We used ANOVAs to evaluate differences in the average number of vertical left-paw digit movements ≥5 px on training and baseline trials per day, and for the average number of vertical right-paw digit movements > 10 px on training and baseline trials per day.

Animals were reinforced based on the average movement of all four digits of their paws. However, we also investigated whether the movement of each individual digit aligned with this average movement. As a follow-up analysis, we evaluated the number of left paw digit movements ≥5 pixels (Fig. 6*A*) as well as the number of right paw digit movements >10 pixels (the maximum criterion for right paw movement to receive a reward) for each digit individually (Fig. 6*B*). Overall, we found a similar pattern of vertical movements across each digit to the mean movement of each paw (Fig. 5), suggesting that individual digits could potentially also be useful as targets for behavioral reinforcement. Additionally, we conducted ANOVAs for each digit on training and baseline trials, and found no significant difference between digits on the number of movements above criterion on baseline or training trials on each day, on either forepaw.

**Figure 7.**
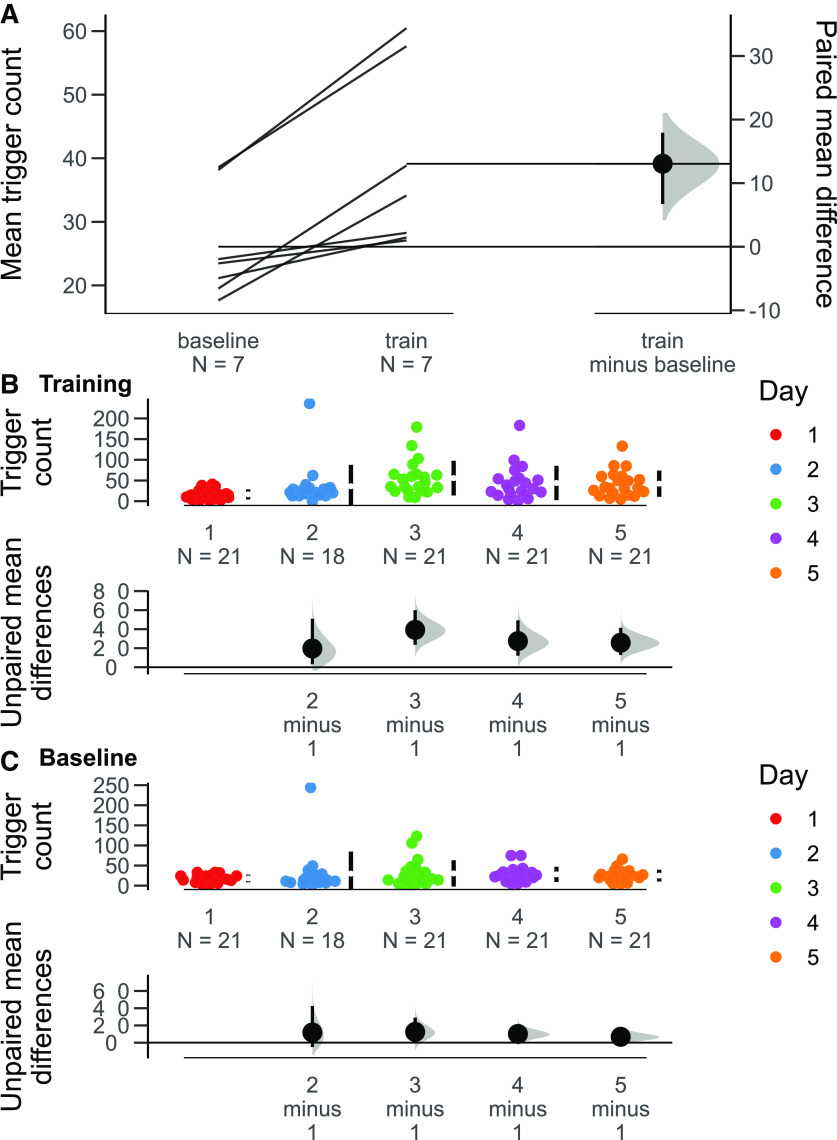
Estimation statistics for the mean difference in trigger count between training and baseline trials on each day and between training trials on each day. ***A***) Slopegraph of mean difference in trigger count by animal between training and baseline trials, and the bootstrapped 95% confidence interval around the mean difference. ***B***) The bootstrapped 95% confidence intervals around the mean differences in trigger count on training trials on each day minus the trigger count on training trials on day 1. ***C***) The bootstrapped 95% confidence intervals around the mean differences in trigger count on baseline trials on each day minus the trigger count on baseline trials on day 1.

As an alternative analysis that does not rely on frequentist statistics, we used the Data Analysis with Bootstrap Estimation in R (dabestr) package (Ho et al., 2019), recommended by Calin-Jageman and Cumming (2019), to conduct a set of estimation statistical analyses. Bootstrap resampling is a robust statistical method that allows for more precise conclusions about patterns of data (Calin-Jageman and Cumming, 2019). Our analyses present the bootstrapped 95% confidence interval surrounding the mean difference between the number of above-threshold movements on baseline and training trials on each day (Fig. 7*A*); the mean difference between the number of number of above-threshold movements during training trials on each day to training trials on the first day (Fig. 7*B*); and the corresponding mean difference comparisons for baseline trials on each day (Fig. 7*C*). The 95% confidence interval for the mean difference between the number of above-threshold movements on baseline and training trials on each day does not cross 0 (Fig. 7*A*). As such, we can say that each animal makes more above-threshold forepaw movements during training trials compared with baseline trials. Furthermore, the 95% confidence intervals for the mean difference between the number of above-threshold movements during training trials on each day to training trials on the first day do not cross 0 for training trials for days 3, 4, and 5 (Fig. 7*B*). Therefore, we can also say that the animals overall make more above-threshold forepaw movements after 2 d of training (Fig. 7*B*) while not making more of these movements on progressive baseline trials (Fig. 7*C*).

## Discussion

We demonstrate a robust real-time tracking and feedback paradigm implemented in Python that can deliver feedback based on individual body part movement with a short delay between movement initiation and LED illumination. Generally, we observed that this delay resulted in the LED being illuminated while the movement that triggered the LED was still in progress (Fig. 3*B*,*C*). This relatively fast feedback based on specific body movements in near real time suggests that our interface could be adapted to provide any type of feedback that is driven by a GPIO signal, including delivery of a water reward or an optogenetic pulse. Additionally, our feedback system, given a robust behavioral model, enables sensitivity to the movements of a single body part, as demonstrated by the system delivering feedback when the left paw moves independently of the right paw. Last, we demonstrate the ability of our system to condition and reinforce user-defined behaviors using a water restriction paradigm.

### Real-time feedback system

Our conclusion is that input frame rates of 200 Hz are optimal for analysis and feedback generation. Increased work on code stability may enable the use of higher-input frame rates, allowing for the measurement of more rapid behaviors. The difference between the input frame rate—the frame rate set on the camera—and the output frame rate—the rate at which frames were processed by our system—likely differs because of the added computational load that DeepLabCut places on the acquisition and analysis system. The most significant factors affecting this frame rate appeared to be (1) the method by which we saved the labeled frames (saving frames asynchronously, as opposed to in the same thread as the threshold computations, provided an improvement of 20–50 Hz at all frame rates tested); and (2) the capabilities of the USB port to which the camera was connected (connecting the camera to a faster USB 3.0 port provided an improvement of 20–50 Hz at all frame rates tested). We were also able to minimize this computational load by running the DeepLabCut pose estimation framework while not saving the outputs for a period of 10 s at the beginning of each trial (Fig. 3*D*). We believe that providing this buffer period has the effect of allowing Python libraries and the complex neural network architecture of DeepLabCut to load.

A number of factors potentially affected the quality of the tracking. First, deviations from the lighting conditions of the videos on which we trained our models occasionally resulted in the tracking of spurious body parts (e.g., the ear) or arbitrary points. In particular, regions of the video with high contrast relative to the intended body parts occasionally became the focus for tracking, especially when lighting conditions were incorrect. This may be a function of how the scoremap calculations that are involved in pose estimation are conducted within DeepLabCut (Mathis et al., 2018). Some lighting changes between the training data and the streamed video were inevitable; we attempted to mitigate these changes by keeping the location of the mouse and the lighting in the room where the experiments took place the same as in the training videos. However, by training our behavioral model for >30,000 iterations across a video dataset that encompassed a wide variety of behaviors, our model became very accurate in distinguishing one paw from the other without the need for additional postprocessing. In fact, a high level of accuracy was generally maintained even during grooming behaviors, when the forms of the paws were obscured. This suggests that variations in camera setup and lighting conditions can be compensated for with a well labeled and trained behavioral model. Second, hardware limitations may also have contributed to increases in the delay. Although our camera was connected to a USB 3.0 port, we conducted a small number of trials (*N* = 4) with a different, faster USB 3.0 port on the computer to compare performance with each port on the computer. Although trials in which the camera was connected to this faster port produced a higher output frame rate for each level of the input frame rate, this solution resulted in a less stable frame rate, leading to much higher delays at frame rates >180 Hz. Third, although the pyftdi library provides a low-level interface between a computer and an LED, the breakout board is connected via USB 2.0, which presents a hardware bottleneck compared with the USB 3.0 technology used for our camera. However, the delay between the movement trigger and the LED activation (measured as the difference in time between the computer time stamp recorded when the left paw movement criterion was reached and the time stamp recorded when the signal was sent to turn on the LED) was very small (mean* *=* *0.31 ms, SD* *=* *0.13 ms) and therefore effectively inconsequential to feedback delivery. Additionally, the mice engaged in grooming behaviors during a number of trials (as discussed earlier). However, while this presented a challenge to the tracking accuracy of our model—as grooming behaviors were not greatly represented in the training dataset—both the tracking accuracy and the behavioral feedback remained robust even during these periods of grooming.

We should caution that the method by which accuracy is quantified (the TensorFlow sigmoid function, which reflects a comparison between the video stream of the mouse and the input data provided to the DeepLabCut movement-tracking model) is not a ground-truth measure of accuracy. However, this method of quantifying the tracking accuracy has been validated against ground-truth human ratings by the developers of DeepLabCut (Mathis et al., 2018). Further progress in increasing the operational speed of DeepLabCut can likely be made by streamlining the lower-level analytical operations of DeepLabCut and the threading strategy used. Because the unstable frame throughput rate in the first 10 s of each trial tended to lead to large delays between frame acquisition and LED illumination early in the recording session, we note that a 10 s buffer period (Fig. 3), during which DeepLabCut conducted pose estimation while no data were saved, was generally necessary to allow the frame throughput rate of our system to stabilize. Additionally, at high-input frame rates (generally >200 Hz), at the time when the system began to save the data (after the 10 s buffer), a number of frames would be dropped during a period of ∼1 s after data saving began. This period of time is the transition period from data not being recorded to the initiation of data recording, which likely causes a spike in computational load that stabilizes within 1 s. We attempted to minimize the number of dropped frames in this period by adjusting the wait period after the end of the 10 s buffering period and before the initiation of data recording. A 100 ms wait period minimized the number of frames dropped during the transition period. We quantified the number of dropped frames by counting the number of data placeholders in our data structure that lacked data; placeholder rows that lacked data were presumed to be dropped frames. If a movement occurred during this period of frame drops, the delay was generally well above average. However, we found that this frame drop generally has little bearing on the delay for the rest of the recording session. Additionally, as we have time-stamp data for each frame start, behavioral trigger, and LED/water release time, we do not rely on the number of frames in a given trial for analysis.

The most critical consequence of dropped frames would be inappropriate timing for the delivery of feedback to the animal. To address this issue, our system makes a decision about whether or not to provide feedback based on two frames of data that are consecutively received from the camera are analyzed together. If a frame is dropped from the camera, these two frames may not be chronologically consecutive. However, our stable frame rate suggests that frames are dropped at a consistent rate (Fig. 3*E*). Specifically, the SD of the time difference between frames was very low (SD = 4.16 ms) across all *N* = 806,153 batches of two frames collected on training and baseline trials outside of the buffer period. According to the Nyquist–Shannon sampling theorem (Shannon, 1949), to capture all the behaviors of the animal, our frame rate would need to be at least double the frequency of the fastest movement that we are sampling. Given that our average frame rate for our second study was 65.59 Hz, we would be able to sample movements at frequencies of up to 32.80 Hz. In a study of mouse locomotion and whisking movement, the maximum stride frequency attained by the mouse was 3–4 Hz, and the peak whisking frequency was 15–20 Hz (Sofroniew et al., 2014). These movements are among the fastest that a mouse can perform—faster than the forepaw movements that we measured in our study—and yet they would theoretically be detectable by our system at the output frame rate of ∼65 Hz given that they are <32.80 Hz. As such, despite the frame loss, our paradigm should be able to capture a wide variety of desired forepaw movements.

Further optimizations to the code would require deeper investigation into the most computationally intensive aspects of DeepLabCut; an especially important area to focus on would be parallelization of pose estimation operations in DeepLabCut. The occasional instabilities of the frame rate in our program, which necessitated the addition of the buffer period, may suggest that DeepLabCut prioritizes fast processing of frames over processing these frames at a stable rate, especially in the first few seconds of the operation of the code. These instabilities may also arise from the creation of a large number of computational threads with no corresponding destruction of finished threads at the start of the task.

We have demonstrated the applicability of our system to tracking forepaw movements in head-fixed mice. However, our software paradigm could be adapted to track and reinforce a variety of different behaviors in near real time (e.g., reaching movements, running on a transparent belt, reaching for pellets or levers). This would entail training a model to track these behaviors using the user-friendly interface of DeepLabCut, then making minor modifications to conditional statements in our code to adjust the threshold for movement to be reinforced. In its current state, our system can drive any output that can be triggered by a GPIO logic signal, be it an LED flash, water pump activation, food pellet release, or electrode stimulation. While we have focused only on head-fixed behaviors, the video-based nature of our system means that the animal needs not be head-fixed for accurate movement tracking and reinforcement to occur. The only requirement is that the model tracking the behavior of the animal be well labeled and trained so as to accurately capture a variety of movement dynamics. One challenge that must be considered in adapting the approach to a freely moving animal would be ensuring that the target body parts can be reliably tracked and are not occluded as the animal transitions to various view angles.

Our motor movement tracking and feedback paradigm could enable new forms of noninvasive closed-loop feedback work that uses motor movements in place of (or augmenting) neural recordings to train animals on a task. Further directions for this research may combine movement tracking with two-photon microscopy to investigate whether DeepLabCut can be used to condition motor behaviors in mice through closed-loop feedback in near-real time, with the potential goal of understanding and localizing motor memory (Fu et al., 2012; Gilad et al., 2018). Additionally, passing our pose estimation through a predefined behavioral state space (Berman et al., 2014) may enable us to evaluate and provide feedback based on more complex behaviors, opening the door to real-time reinforcement of sophisticated motor activities. We have released the code for implementing our movement tracking and feedback system as a Python script that integrates with DeepLabCut version 2 or later. With minor modifications to the logic by which the feedback is triggered in the code, our system could be adapted to track virtually any user-defined body part or animal that is defined in a DeepLabCut model. Future developments of this system would benefit from the inclusion of a more nuanced behavioral classifier that is trained to identify and trigger feedback based on complex behavioral dynamics and movement trajectories. Such a classifier could open the door to the rapid training of even complex behaviors in animals.

### Validation through behavioral task

In order to demonstrate the utility of our real-time feedback system in a real-world context, we used our system to automatically reinforce water-restricted mice for making forelimb movements. Mice made significantly more left paw movements during training trials on later training days than on the first day (Figs. 5*A*, 7*B*), while the number of left paw movements remained stable across all baseline trials (Figs. 5*A*, 7*C*). These movements were generally not accompanied by large right paw movements (Fig. 5*B*), demonstrating the selectivity of our system for reinforcing the movement of a specific paw. Furthermore, these movements were deployed consistently across the whole paw (Fig. 6). Additionally, all mice made more movements satisfying the criterion on training trials (where they were reinforced) than on baseline trials (Fig. 7*A*). Conceivably, it may be easier to train the mouse to make a highly stereotyped behavior as opposed to merely giving the animal a specific criterion for the extent of their movement. The fact that our system was nevertheless able to reinforce a wide range of movements allows for greater training flexibility than a conventional behavioral reinforcement paradigm such as a lever press in an operant chamber. With minor modifications to the Python script driving the behavioral feedback, we could set up our system to reinforce a wide variety of user-defined behaviors in many different animals. For example, our system could be used to efficiently train a rodent in a stereotyped motor behavior to promote the recovery of motor function poststroke.

### Conclusion

This project could form the basis for future work on closed-loop behavioral reinforcement systems that include brain stimulation (Paz et al., 2013; Prsa et al., 2017) and could be used to explore the basis of various movement-related and somatosensory activities in the brain (Stubblefield et al., 2013). Such exploratory research could contribute to more advanced and effective biofeedback that leverages both neural and non-neural movement data, adding greater diversity to the types of information signals. With additional reductions to the latency of our system, this work could inform brain–machine interfaces, enabling direct reinforcement of animal behavior based on movement dynamics.

## Synthesis

Reviewing Editor: Quentin Pittman, University of Calgary Decisions are customarily a result of the Reviewing Editor and the peer reviewers coming together and discussing their recommendations until a consensus is reached. When revisions are invited, a fact-based synthesis statement explaining their decision and outlining what is needed to prepare a revision will be listed below. The following reviewer(s) agreed to reveal their identity: Tamas Fuzesi.

The adaptation of DeepLabCut allowing closed loop feedback systems is interesting and potentially highly useful for an eventual understanding of neural plasticity and learning behavior. However the reviewers agree that it is limited at the current time to the head fixed animal . Thus we recommend:

1. The title should be modified to indicate that the method is applied to head fixed mice and is applicable to paw movement s only. Recommendation: Real-time selective markerless tracking of forepaws of head fixed mice using deep neural networks

2. You discuss applicability and challenges to adapting the approach to more naturalistic behaviors in freely moving animals, for example in pellet retrieval, reaching tasks or in moving on a transparent belt.

3. While the authors report it transparently, the rate of dropped frames is quite concerning. Also, the authors mention that the first 10 seconds of the recordings were discarded “to allow the framerate and DeepLabCut’s computations to stabilize”. What happened in this first 10 seconds? Why did it stabilize after 10 seconds? My main concerns here is if this solution is adopted by other labs certainly with some variations in the hardware, how can they be sure that 200 Hz framerate will provide an “acceptable” drop in frame number? Or if 10 seconds of recording is enough to stabilize frame rate

5. The authors showed in the first study that the system detected the left paw movement without the right paw movement at the same frame. However, in the second study, the movement of the right paw might frequently occur immediately after the left paw movement. If so, the movement determined by the experimenter was not specifically reinforced. Why was the threshold for the right paw 10 pixels although that for the left paw was 5 pixels? In addition to figure 5, the authors should show the count of the right paw that crossed over the threshold of 10 pixels around the triggered frame.

Minor suggestions:

1.Although four individual digits in each paw were tracked, only the averaged trace of them was used. It would be better to show the trace of each digit and discuss whether the above-threshold movement of each digit can be robustly detected.

2. In figure 4C, both SEMs of traces and the trigger timing are shown in grey. It would be better to use different colors.

3. Statements linking this real time tracking approach to a brain-machine interface sounds a bit too ambitious at this point. Especially if the delay between when the above-threshold movement was received and the time at which the command was sent to activate the LED trigger varies 30-40 ms in a trial.

4. In Fig3E, why are the dots (the delays) are seemingly discreetly distributed? Looks like there are only 6 different set delay times. Is that true? If yes, why?

5. The manuscript could benefit a lot from a thorough proof reading. There are repeated sentences (i.e. line 13-15 and 20-22; line 189-190 and 192-195) and clear mistakes (i.e. line 11: “We adapt DeepLabCut for real-time tracking as an open-source package we term DeepLabCut”).

6. Some example videos of tracking and the training would help a lot to appreciate the quality of the author’s solution.

7. Tables 1 and 2 are described as summary statistics. They are not. Rather they are data tables. Please include a title for Table 1.

8. Fig 5; please indicate in the legend which statistical tests were used. In addition, the preference of eNeuro is that each data point should also be plotted, superimposed upon the bars. You may also consider using estimation statistical tests as suggested in the instructions to authors.
